# Recurrent sick leave and resignation rates among female cancer survivors after return to work: the Japan sickness absence and return to work (J-SAR) study

**DOI:** 10.1186/s12889-019-7509-3

**Published:** 2019-09-11

**Authors:** Motoki Endo, Yasuo Haruyama, Go Muto, Yuya Imai, Kiyomi Mitsui, Tetsuya Mizoue, Hiroo Wada, Gen Kobashi, Takeshi Tanigawa

**Affiliations:** 1 Department of Public Health, Juntendo University Faculty of Medicine, 2-1-1 Hongo, Bunkyo-ku, Tokyo, 113-8421 Japan; 2 Department of Public Health, Dokkyo University School of Medicine, Tochigi, Japan; 3 Department of Epidemiology and Environmental Health, Juntendo University Faculty of Medicine, Tokyo, Japan; 4 Department of Hygiene, Public Health and Preventive Medicine, Showa University, Tokyo, Japan; 5 Department of Epidemiology and Prevention, Center for Clinical Sciences, National Center for Global Health and Medicine, Tokyo, Japan

**Keywords:** Cancer survivors, Return to work (RTW), Recurrent sick leave, Resignation

## Abstract

**Background:**

To date, there have not been any workforce-based Japanese cohort studies investigating work sustainability after return to work (RTW). The objective of this study was to investigate the post-RTW cumulative recurrent sick leave rate and cumulative resignation rate among female cancer survivors.

**Methods:**

Among Japanese employees who were registered in the Japan sickness absence and return to work (J-SAR) study, the subjects were those female employees who returned to work after sick leave due to newly clinically diagnosed cancer (C01-C99; ICD-10), based on a physician’s certificate, between 2000 and 2011. The last day of the follow-up period was December 31, 2012. The recurrent sickness leave rate and resignation rate were calculated using competing risk survival analysis.

**Results:**

Of 223 cancer survivors, 61 took further physician-certified sick leave after their RTW. The median duration of the post-RTW work period among all cancer survivors was 10.6 years. The work continuance rates of the female cancer survivors were 83.2 and 60.4% at 1 and 5 years after they returned to work, respectively. There was a steep reduction in the work continuance rate during the first post-RTW year. There were considerable differences in the work continuance rate according to the primary cancer site. Cumulative recurrent sick leave rates of 11.8 and 28.9% were seen at 1 and 5 years after the subjects returned to work. The cumulative resignation rate was 5.0 and 10.7% at 1 and 5 years after the subjects returned to work. Most recurrent sick leave occurred in the first year after the subjects returned to work, followed by the second year.

**Conclusions:**

Sixty percent of female cancer survivors were still working at 5 years after returning to work, although the work continuance rates for different types of cancer varied significantly.

## Background

According to the statistics of the Ministry of Internal Affairs and Communication, Japan (2010), the working age population in Japan includes 81.3 million people; however, it is estimated that it will decrease in size by almost 50% in the next 50 years [1]. Currently, Japan is transitioning to a society where women, senior citizens, and foreigners will be indispensable to the employment market [2]. In many developed countries, the number of cancer patients in the working population is increasing, and the probability that workers will suffer from cancer is also rising [2–4]. In Japan, these phenomena are caused by the following four factors: the first factor is the increase in the number of senior citizens that are still working. It has been reported that some workers in their 60s develop cancer after becoming temporary workers after their mandatory retirement at 60 years old [1]. The second factor is an increase in the number of working women. In fact, the number of double-income households (in which both the husband and wife work) markedly exceeds the number of households that include full-time housewives [1]. The third factor is the increasing incidence of cancer among females, especially breast cancer, and the younger age of onset of cervical cancer [5]. The fourth factor is that the prognosis of cancer patients has improved due to the development of treatments that place less burden on the body [6].

As cancer survival rates have improved due to recent advances in cancer treatment, more cancer survivors are able to resume their everyday lives and return to work (RTW) during or after treatment [3, 7, 8]. While cancer is more likely to occur among the elderly, breast cancer and uterine cancer are common in people of working age [9].

In February 2016, the “guidelines for the support of therapy and working life in the Japanese workforce” were published by the Japanese Ministry of Health, Labour and Welfare [10]. As a result of the revision of the Cancer Control Act in December 2016, companies in Japan are required to support the reinstatement of employees with cancer [11].

While returning to work would seem to indicate that a cancer survivor has completely recovered from their illness and the adverse effects of treatment, it actually only demonstrates that their ability to perform their job has recovered to a level where they can be reinstated. After returning to work, cancer survivors often encounter difficulties associated with the balance between the demands of treatment and recovery and the requirements of the workplace [12–14]. Some cancer survivors continue to receive chemotherapy, whereas others suffer from cancer-related fatigue (CrF), pain, functional impairment, and/or psychological distress [15, 16]. Working after returning to work is extremely challenging for cancer patients, especially with regard to physical and mental health [17, 18].

Although a number of studies of cancer survivors have been carried out in Europe and the US, there have not been any workforce-based Japanese cohort studies investigating the frequency of recurrent sick leave or resignations among female cancer survivors that they RTW [15, 19–22]. It is very important to clarify the work sustainability of female cancer survivors that RTW, as females in their 20s to 40s are more likely to suffer from cancer than their male counterparts [5].

The objective of this study was to investigate the cumulative recurrent sick leave rate and cumulative resignation rate after Japanese female cancer survivors RTW and to analyze predictors of recurrent sick leave, resignation between different cancers. This study is the first cohort study to examine the work sustainability of cancer-affected female employees in Japan.

## Methods

### Subjects

The Japan sickness absence and return to work study (J-SAR study) was a retrospective, observational cohort study conducted in Japan. The J-SAR study analyzed data regarding sick leave that were registered in the health data system of a private occupational health center. This occupational health center had signed occupational health service with the following companies: regional, international telecommunication, big data and mobile services (installing and maintaining telephones, faxes, telegrams, internet systems, cell phones), logistics, energy, and construction etc., as described elsewhere [15, 23–28]. In 2000, about 68,000 employees were working for these companies on a full-time basis. In this study, we extracted the data of all employees who had taken sick leave due to newly diagnosed cancer between January 1st, 2000, and December 31st, 2011.

### Sickness absence system

In Japan, there is no law insuring sickness absence for employees who are not able to work. To our knowledge, almost all large Japanese companies have their own sickness insurance system for employees. The time limit for sickness absence varies depending on the company. In the Japanese sickness absence system, part-time sickness absence combined with part-time work is not so common. The fact is that many small and medium-sized enterprises in Japan do not have such an established sickness insurance system. We guess that cancer survivors who work at small and medium-sized enterprises have no choice but to quit because of their companies’ economic circumstances, among other factors. On the other hand, this Japanese company group has more improved sickness absence system and RTW system than other companies, as described in our previous studies [15, 23–28].

### Inclusion criteria

The subject inclusion criteria for this study were as follows: female employees, aged 18 to 60 years, who returned to work after their first period of sick leave due to clinically diagnosed cancer (C01-C99; ICD-10) based on a physician’s certificate between January 1st, 2000, and December 31st, 2011. Two hundred forty-five female employees had sick leave due to cancer newly during the duration, as described in our previous study [15]. Of these female cancer survivors, 223 returned to work. Based on these inclusion criteria, the first period of sick leave was not a period of recurrent sick leave because employees who had suffered previous episodes of sick leave due to cancer before 31 December 1999 were not included in this study. The medical ethics committee of Juntendo University informed us that ethical approval was not required because the data were existing data that were anonymous and impossible to concatenate, and no associated correspondence table exists.

### Statistical analysis

The work continuance rate was estimated via Kaplan-Meier survival analysis. Numbers of person-days were calculated based on the follow-up period. The day used to measure the beginning of the follow-up period was the first day of the subject’s RTW after their sick leave due to cancer. In this study, the data were censored at the end of the follow-up period (December 31, 2012) or the day of retirement (March 31 of the year that the subject became 60 years old), whichever came first. The event day for this analysis was the first day of recurrent sick leave due to any physician-certified illness or the day of resignation before retirement. Deaths were analyzed as “recurrent sick leave”. During the follow-up period, 12 female employees died after RTW.

Cumulative recurrent sick leave and resignation rates were calculated via competing risk survival analysis [29]. A survival analysis with competing risks was performed using EZR, which is statistical analysis software provided by CRAN (The Comprehensive R Archive Network) [30].

## Results

In total, 223 female employees returned to work after their first period of sick leave due to cancer. The subjects’ characteristics are shown in Table 1. Among these employees, breast cancer was the most common type of cancer (ICD-10, C50; *n* = 90). The second most common type of cancer was female genital malignancy, which included cancer of the uterus (*n* = 47) and ovarian cancer (*n* = 17). The third most common type of cancer was gastric cancer (*n* = 18), followed by lung cancer (*n* = 16). The other types of cancer (*n* = 35) seen among the employees included colon cancer (*n* = 5) and esophageal cancer (*n* = 3). The subjects’ mean age on the initial day of their sick leave was 48.2 years old. Just after returning to work, 185 cancer survivors (83.0% of RTW employees) had their work schedules reduced based on OP certificates, and 38 cancer survivors were able to work full-time.

**Table 1:** Basic characteristics of the subjects in this study

	n	Mean age (±SD)	Urban / Rural (n)	Desk / manual worker (n)	1. Experienced RSA (n) (%)	2. Resigned (n) (%)	3. Censored (n)(%)	1-year work survival rate (%)	5-year work survival rate (%)	Median time to work disability (years)
Cancer site										
Breast cancer	90	48.4 ± 6.6	68/ 22	89/ 1	22 (24.4%)	9 (10.0%)	59 (65.6%)	82.9	63.4	N.A.
Female genitals	64	46.6 ± 7.3	48/ 16	64/ 0	19 (29.7%)	1 (1.6%)	44 (68.8%)	87.3	67.8	N.A.
Gastric cancer	18	52.4 ± 5.1	11/ 7	17/ 1	2 (11.1%)	3 (16.7%)	13 (72.2%)	83.3	63.1	N.A.
Lung cancer	16	52.1 ± 4.5	16/ 0	15/ 1	5 (31.3%)	7 (43.8%)	4 (25.0%)	75.0	31.3	3.0
Other cancer	35	46.4 ± 8.4	29/ 6	31/ 1	13 (37.1%)	2 (5.7%)	20 (57.1%)	80.0	55.1	N.A.
Total Stroke	223	48.2 ± 7.1	172/ 51	216/ 4	61 (27.3%)	22 (9.9%)	140 (62.8%)	83.2	60.4	10.6

Female genital malignancy included cancer of the uterus (*n* = 47) and ovarian cancer (*n* = 17)

Other cancer included colon cancer (*n* = 5) and esophageal cancer (*n* = 3)

*RSA* Recurrent sick leave

After returning to work, 61 subjects (27.3%) experienced further periods of sick leave (recurrent sick leave) due to medical reasons, which were certified by their physicians. Of these 61 employees, 43 experienced recurrent sick leave due to their original cancer, 4 developed new types of cancer (other cancer), 4 suffered from common mental disorders, and 3 suffered fractures. Twenty-two cancer survivors resigned before reaching retirement age (60 years). Among all cancer survivors, the median duration of the period of work after the subjects initially returned to work was 10.6 years. During the follow-up period, 12 female employees died after the day of RTW. Of the 12 deaths, 9 employees died within the period of recurrent sick leave.

### Work continuance rates, recurrent sick leave, and reassignment after returning to work

**Fig. 1:**
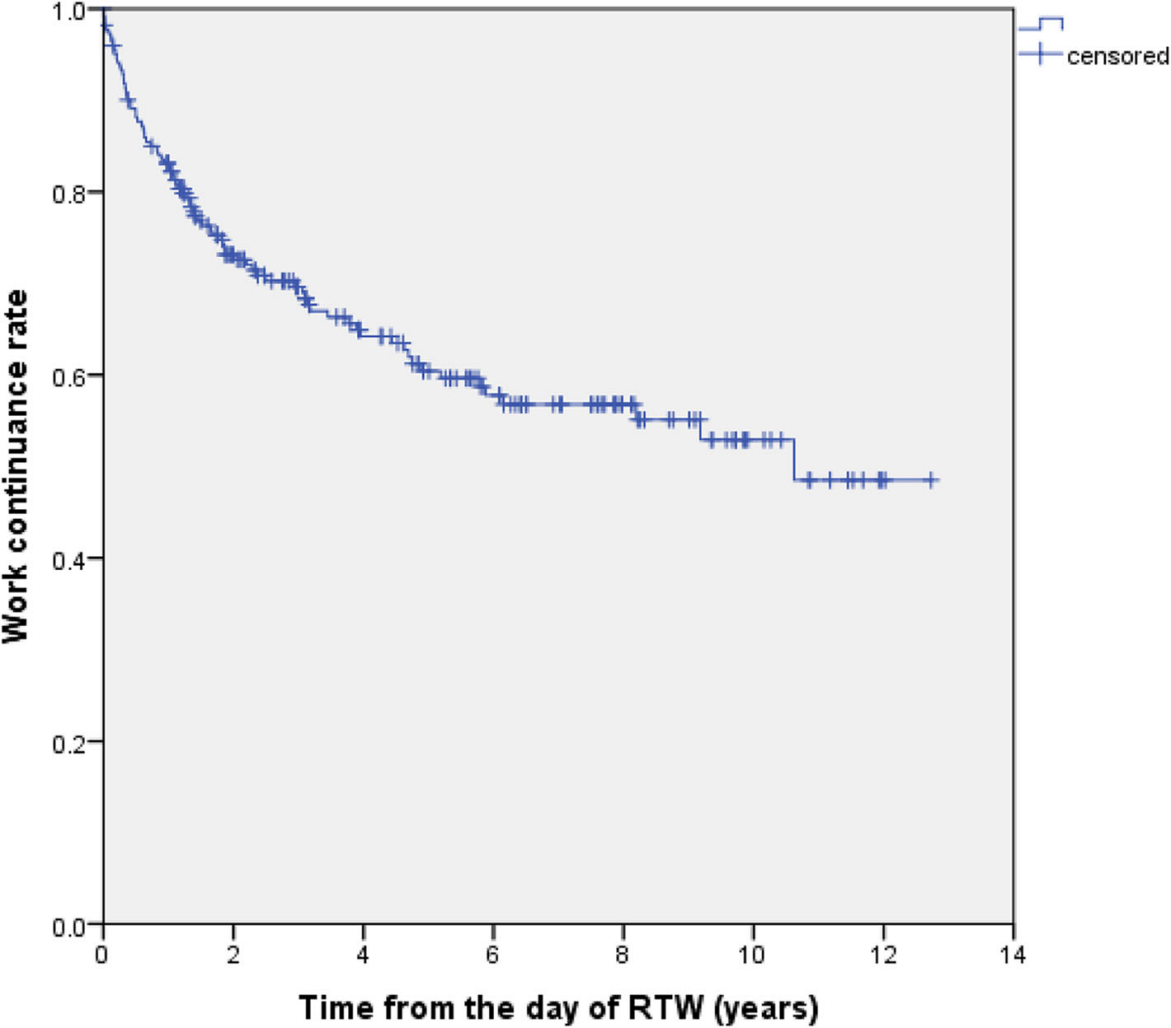
Work continuance rate from the day of RTW according to Kaplan-Meier survival analysis

**Fig. 2:**
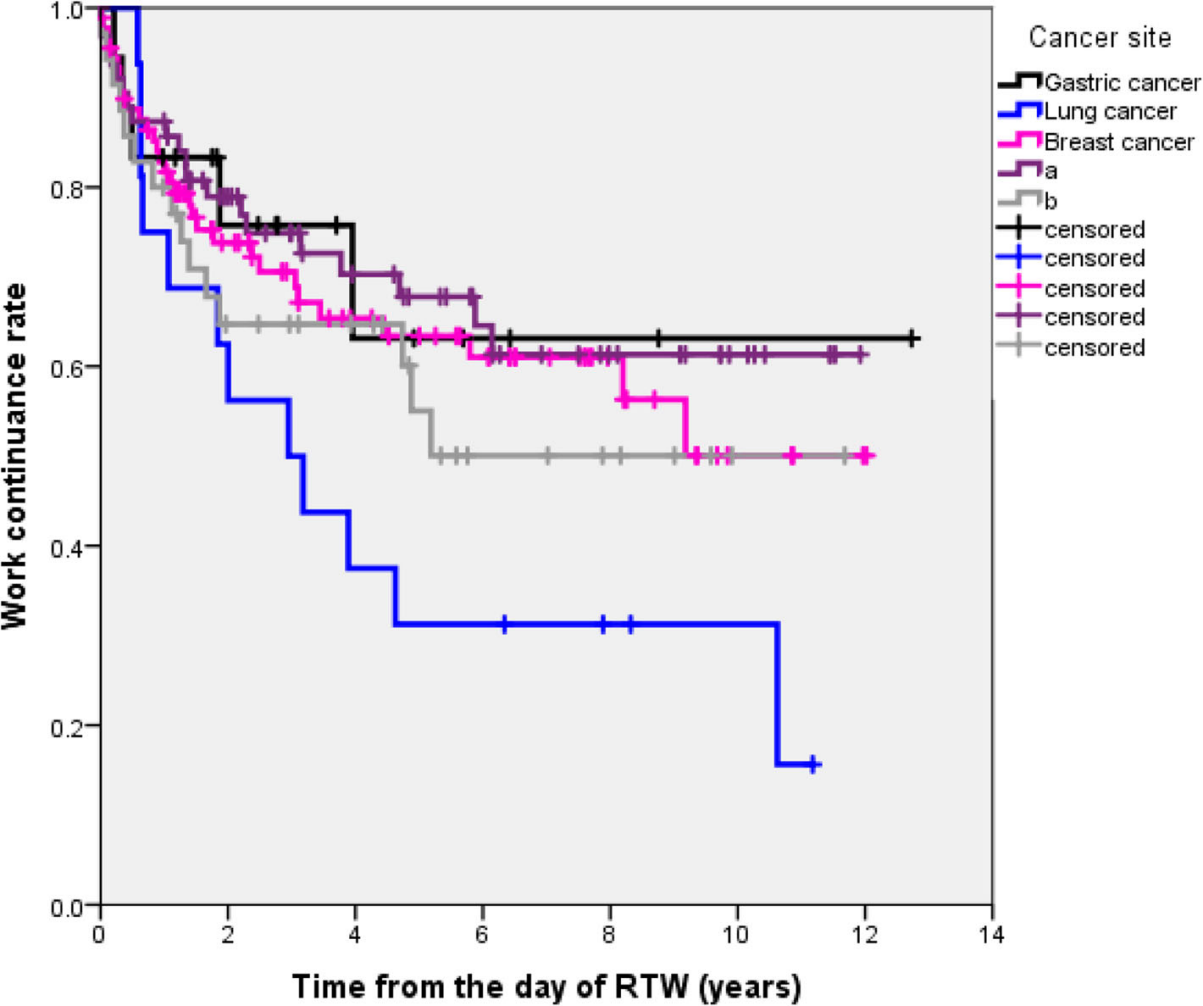
Work continuance rate from the day of RTW stratified by cancer site. a Female genital malignancy included cancer of the uterus (*n* = 47) and ovarian cancer (*n* = 17). b Other cancer included colon cancer (*n* = 5) and esophageal cancer (*n* = 3)

According to Kaplan-Meier survival estimates, the work continuance rate of the female cancer survivors was 88.2% at 6 months, 83.2% at 1 year, 73.1% at 2 years, 69.6% at 3 years, 64.2% at 4 years, and 60.4% at 5 years after they initially returned to work, as shown in Fig. 1. Figure 2 shows that there was a steep decrease in the work continuance rate during the first year after the subjects returned to work. It should be noted that the work continuance rate varied markedly according to the primary cancer site (Fig. 2). However, there were no statistically significant difference between different cancers, as for recurrent sick leave and resignation.

**Fig. 3:**
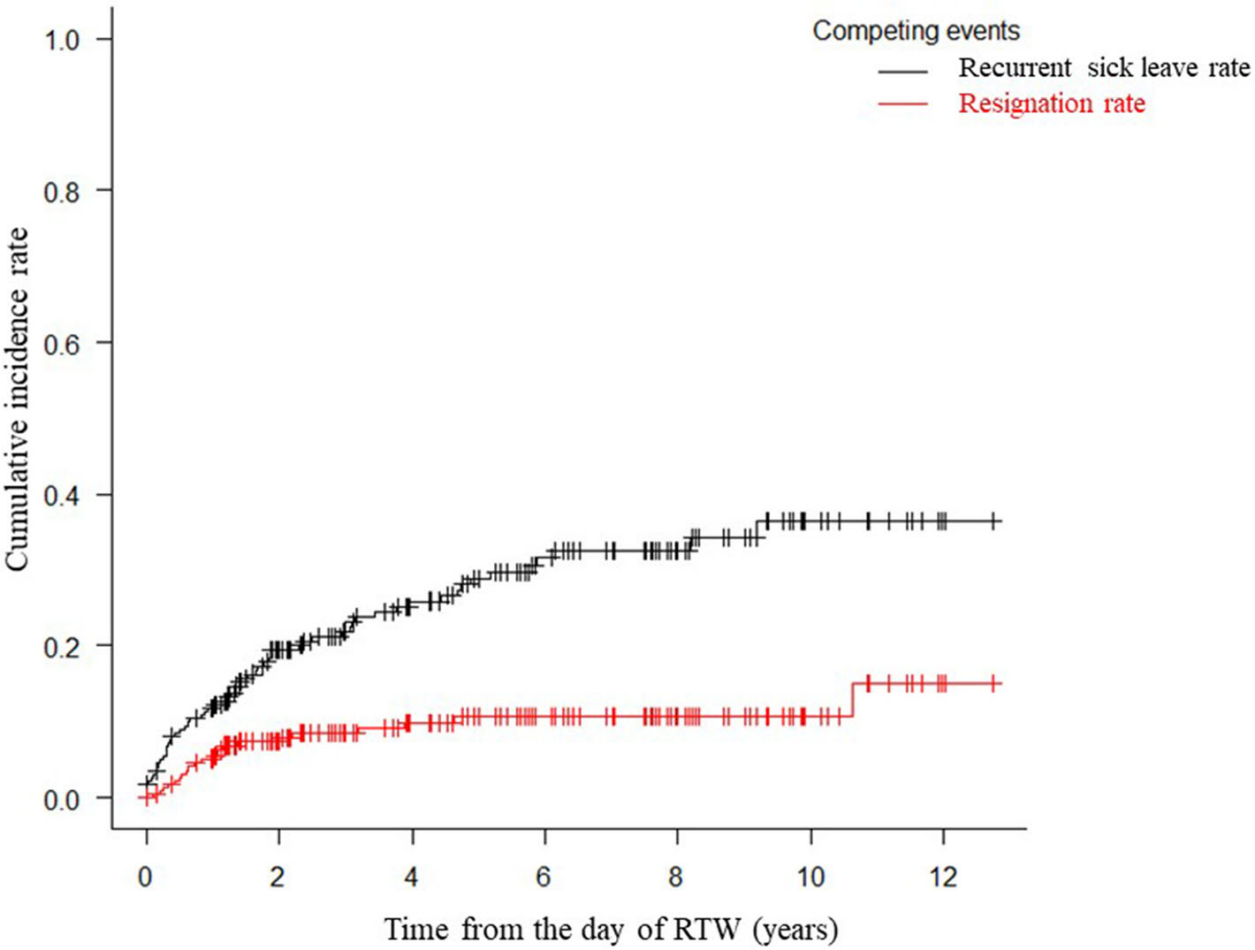
Post-RTW recurrent sick leave and resignation rates according to competing risk analysis

The work continuance rate of the subjects with breast cancer patients was 82.9% at 1 year, 73.8% at 2 years, and 63.4% at 5 years. The work continuance rate of the subjects with lung cancer was 70.5% at 1 year, 62.5% at 2 years, and 31.3% at 5 years, and these values were lower than those of the other types of cancer. As shown in Fig. 3, in the survival analysis of competing risks, the cumulative recurrent sick leave rate was shown to be 9.1% at 6 months, 11.8% at 1 year, 19.5% at 2 years, 21.9% at 3 years, 25.9% at 4 years, and 28.9% at 5 years after the subjects returned to work. The cumulative resignation rate was 2.7% at 6 months, 5.0% at 1 year, 7.4% at 2 years, 8.5% at 3 years, 9.9% at 4 years, and 10.7% at 5 years after the subjects returned to work, as shown in Fig. 3.

## Discussion

As far as we know, this is the first longitudinal cohort study to investigate the work sustainability of female cancer survivors after they RTW. We found that about 60% of female cancer survivors were still working at 5 years after their RTW, although the work continuance rate differed significantly among different cancer types. In particular, the lung cancer patients exhibited had the lowest work continuance rate. Though there were no statistically significant difference between different cancers, as for recurrent sick leave and resignation, that could be caused by the rather small sample in this study.

The main work-related problems experienced by female cancer survivors after they RTW are as follows: the first is recurrent sick leave due to a physician-certified medical reason (including death). As shown in Fig. 2, we found that recurrent sick leave most commonly occurred in the first year after the cancer survivors returned to work, followed by the second year. Of the female cancer survivors who required recurrent sick leave (*n* = 61), 25.0, 32.4, and 53.6% required such leave within 6 months, 1 year, and 2 years of their RTW, respectively. The competing risk curve for recurrent sick leave plateaus at 5 years after a cancer survivor returns to work, and hence, is very similar in shape to the Kaplan-Meier curve of recurrent sick leave due to depression produced in a previous study [26].

The second problem experienced by female cancer survivors is having to resign before they reach retirement age (60 years old). In the present study, the cumulative resignation rate was unexpectedly low. The survival curve of the cumulative resignation rate remained below 10% until 4 years after the subjects returned to work. Of all the post-RTW resignations, 17.9, 33.1, 49.0, 56.2, 65.6, and 70.9% had occurred within 6 months, 1 year, 2 years, 3 years, 4 years, and 5 years after the subjects returned to work. Short et al. reported that about 10% of cancer survivors resigned from their jobs within a 4-year period because of cancer-related factors [31]. Stewart et al. reported that about 12.5% of breast cancer survivors quit their jobs before retirement as a result of cancer [32].

Female cancer survivors’ work continuance rates (60.4%) at 5 years after the RTW day were higher than male cancer survivors (48.5%) [27]. We speculated that the probability of more male cancer survivors who had cancer sites of lower work continuance rates (for example, lung cancer, hepatic cancer, pancreatic cancer, esophageal cancer) was higher than female. As for comparing work continuance rates by cancer sites, the female work continuance rates 63.1% of gastric cancer at 5 years after RTW was almost same as male (62.1%). However, the female work continuance rates of lung cancer (31.3%) at 5 years after RTW were higher than male (12.1%). As the 5-year relative survival rates of lung cancer among female, male cancer survivors were 43.2, 27.0%, respectively, the epidemiology of lung cancer tissue types might affect the difference in the work continuance rates between women and men [9] While only 4 employees in the present study took recurrent sick leave due to mental disorders (certified by psychiatrists), cancer survivors often suffer from more severe anxiety and depression than non-cancer survivors [33]. Cancer survivors might fear the recurrence or progression of their disease, which might act as a strong stressor, even years after the initial diagnosis [34]. While employees with cancer struggle to stay in work, raise their children, and live a normal life, the work sustainability of female cancer survivors tends to be lower than that of male cancer survivors [35]. Gudbergsson et al. reported that female cancer survivors experienced more job-related strain than males [36]. In addition, De Boer et al. reported that it took more time for female cancer survivors to recover their work abilities than male cancer survivors [20]. Our study showed that the work continuance rate of female cancer survivors was higher than that of male cancer survivors, while the survival rate of Japanese female cancer survivors was estimated to be higher than that of Japanese male cancer survivors according to the Japan cancer registry [27, 37]. As for the social support provided to cancer survivors with work-related disabilities, it varies from country to country. In the Netherlands, cancer survivors can apply for a work disability benefit after being on sick leave for 2 years, whereas working Japanese cancer survivors are not supported by similar laws or government recommendations, and so the support available depends on the employee’s company [2, 38]. In a study by Bradley et al., the proportion of cancer survivors that underwent a partial RTW was lower than in the present study (80%) [39]. The female cancer survivors in the current study were supported by OP, who encouraged the use of adaptations to facilitate partial RTW because they worked in large companies. We speculate that, more female cancer survivors would be able to continue their work after RTW, if they are supported by various employment support even in small and medium-sized businesses. This study provides useful epidemiological information about RTW support, especially for cancer patients in small and medium-sized enterprises [15]. However, it should always be noted that studies can differ in terms of their design, methodology, and the social circumstances of their subjects [40, 41].

### Strengths and limitations

As for the strengths of this study, it was the first longitudinal study (it covered a period of > 10 years) to investigate the frequencies of recurrent sick leave and resignation after female cancer survivors RTW. Secondly, the registered sick leave data analyzed in this study were based on physicians’ certificates, which increases the study’s validity.

As for the limitations of this study, the results of this study should be interpreted carefully. First, the patients’ medical files were not available, and so we did not have any information about the treatments the subjects received, the side effects previous treatments induced, or cancer-related symptoms. As a future task, the effects of clinical factors (e.g., surgery, chemotherapy, and radiotherapy) on work sustainability among female cancer survivors should beconsidered. Especially, CrF is known to be one of the most influential factors for work sustainability, caused by surgery, chemotherapy, radiotherapy, and hormone treatment [42–44]. Secondly, all of the study subjects worked at large companies; thus, it might be difficult to generalize the results of this study to small and medium-sized enterprises. Thirdly, data of sick leave and resignation among other workers in the companies were not available. Fourthly, this RTW system in which the company decides who are allowed to work, might include study participants with higher work ability than in systems in which it is up to the employee to decide whether she is fit to work. Fifthly, as resignation after RTW was evaluated as work discontinuation in this study, some subjects stopped working for the company being studied, but continued working elsewhere.

## Conclusion

Sixty percent of female cancer survivors were still working at 5 years after they returned to work, although the work continuance rate differed significantly among the various types of cancer. Recurrent sick leave occurred most frequently in the first year after the subjects returned to work, followed by the second year.

## Data Availability

It is not possible to share J-SAR study data publicly. The datasets analyzed during the current study are not publicly available due to this company group’s request. We had J-SAR study data supporting the results reported in the article. The J-SAR study analyzed data regarding sick leave that were registered in the health data system of a private occupational health center, which belongs to a large-scale Japanese company group. J-SAR study data analyzed during this study are included in these published articles [15, 23–28].

## Abbreviations

CrF: Cancer-related fatigue
J-SAR study: The Japan sickness absence and return to work study
OP: Occupational Physicians
RSA: Recurrent sickness absence
RTW: Return to work
